# DKK1 rescues osteogenic differentiation of mesenchymal stem cells isolated from periodontal ligaments of patients with diabetes mellitus induced periodontitis

**DOI:** 10.1038/srep13142

**Published:** 2015-08-17

**Authors:** Qi Liu, Cheng-Hu Hu, Cui-Hong Zhou, Xiao-Xia Cui, Kun Yang, Chao Deng, Jia-Jia Xia, Yan Wu, Lu-Chuan Liu, Yan Jin

**Affiliations:** 1 Department of stomatology, Daping hospital, Research institute of Field surgery, Third military medical university, Chongqing, 400042, China; 2 State Key Laboratory of Military Stomatology, Center for Tissue Engineering, School of Stomatology, Fourth Military Medical University, Xi’an, Shaanxi 710032, China; 3 Research and Development Center for Tissue Engineering, Fourth Military Medical University, Xi’an, Shaanxi 710032, China; 4 Department of Periodontology, Stomatological hospital, Zunyi medical college, Zunyi, Guizhou 563003, China; 5 Xi’an Institute of Tissue Engineering & Regenerative Medicine, Shaanxi 710032, China; 6 Department of Oral Histology and Pathology, School of Stomatology, Fourth Military Medical University, Xi’an, Shaanxi 710032, China

## Abstract

Multiple studies have shown that diabetes mellitus is an established risk factor for periodontitis. Recently mesenchymal stem cells derived from periodontal ligament (PDLSCs) have been utilized to reconstruct tissues destroyed by chronic inflammation. However, impact of periodontitis with diabetes mellitus on PDLSCs and mechanisms mediating effects of complex microenvironments remain poorly understood. In this study, we found multiple differentiation potential of PDLSCs from chronic periodontitis with diabetes mellitus donors (D-PDLSCs) was damaged significantly. Inhibition of NF-κB signaling could rescue osteogenic potential of PDLSCs from simple chronic periodontitis patients (P-PDLSCs), whereas did not promote D-PDLSCs osteogenesis. In addition, we found expression of DKK1 in D-PDLSCs did not respond to osteogenic signal and decreased osteogenic potential of D-PDLSCs treated with DKK1 could be reversed. To further elucidate different character between P-PDLSCs and D-PDLSCs, we treated PDLSCs with TNF-α and advanced glycation end products (AGEs), and find out AGEs which enhance effect of TNF-α in PDLSCs might mediate special personality of D-PDLSCs. The adverse effect of AGEs in PDLSCs could be reversed when PDLSCs were treated with DKK1. These results suggested DKK1 mediating WNT signaling might be a therapy target to rescue potential of PDLSCs in periodontitis with diabetes mellitus.

Periodontitis is a globally prevalent inflammatory disease which causes the destruction of the periodontal structures (i.e. alveolar bone, periodontal ligament and root cementum) and potentially leads to tooth loss^1^. Periodontal ligament stem cells (PDLSCs), a population of mesenchymal stem cells (MSCs), were recently used to regenerate lost tooth-supporting apparatus^2^. Further, regeneration potential of endogenous PDLSCs was impaired in periodontal ligament with chronic periodontitis^3^. Diabetic mellitus is associated with increased prevalence, severity and progression of periodontitis which is as the “sixth complication” of diabetes^4^. However, characteristics of PDLSCs from periodontitis patients with diabetes are still unknown.

In the past decade there have been considerable advances in our understanding of diabetes and periodontitis. There been a growing awareness that function of advanced glycation end products (AGEs) is necessary to be identified in clinical patients^5^. The expression of receptor for advanced glycation end products (RAGE) is increased in patients with diabetes mellitus, and its activation through interaction with its ligands (AGEs) is the primary concern in the development and progression of other diabetic complications such as periodontitis^6^,^7^,^8^. Blockade of RAGE not only suppresses periodontitis-associated bone loss in diabetic mice, but also decreased generation of the proinflammatory cytokines in gingival tissue^9^. Enhanced RAGE expression in an environment, such as the periodontium of an individual with diabetes mellitus, leads to exaggerated inflammation and impaired repair, which then results in accelerated and severe periodontal destruction^10^,^11^. Additionally, AGEs could attenuated mesenchymal stem cells (MSCs) and prevent tissue repair by inhibiting the maturation of MSCs-derived cells^12^, but the mechanisms mediating AGEs-RAGE interaction were poorly understood.

Recently, RAGE could be as mediator of an interaction between inflammation and oxidative stress through NF-κB signaling^13^. We have previously demonstrated that NF-κB signaling is activated in PDLSCs from periodontitis and blocking NF-κB signaling can rescue osteogenic potential of the cells^14^. In this study, we investigated the role of NF-κB signaling in osteogenesis of PDLSCs from periodontitis patients with diabetes (D-PDLSCs). We found that inhibition of NF-κB signaling in diabetic microenvironments could not attenuate impaired changes of PDLSCs induced by AGEs-RAGE interaction. Additionally, the expression of DKK1 was inhibited in D-PDLSCs, and inhibited WNT signaling by DKK1 could modulate the expression of RAGE and reverse impaired osteogenic potential of D-PDLSCs.

## Results

### Osteogenic or adipogenic potential of P-PDLSCs and D-PDLSCs were impaired

**Figure 1:**
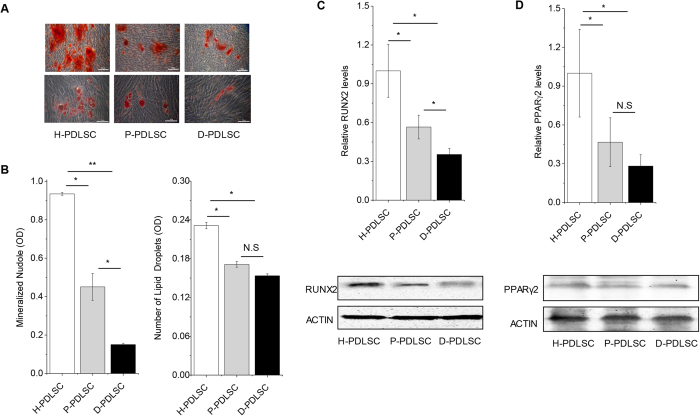
Differentiation potential of mesenchymal stem cells from periodontal ligaments (PDLSCs) was impaired in P-PDLSCs and D-PDLSCs. (**A**) PDLSCs from normal, periodontitis and periodontitis with diabetes mellitus persons were induced to osteogenic differentiation for 28 days or adipogenic differentiation for 21 days. Osteogenic or adipogenic differentiation was separately determined by Alizarin Red S staining or Oil Red O staining, bar = 50 μm. (**B**) Quantification of Alizarin Red staining and Oil red O staining (n = 5). (**C**) Real-time PCR and western blot analysis of the osteoblast marker gene (RUNX2, normalized to β-actin) on day 7 (n = 3). (**D**) Real-time PCR and western blot analysis of the adipogenic marker gene (PPARγ2, normalized to β-actin) on day 3 (n = 3). Data (±SD) are representative of two (**C**,**D**) or three (**B**) independent experiments. Student’s t test was performed to determine statistical significance (*p < 0.05, **p < 0.01).

Consistent with our previous results^15^, osteogenic and adipogenic potential of P-PDLSCs decreased significantly compared with H-PDLSCs (Fig. 1). Although both mesenchymal stem cells produced mineralized extracellular matrices which were positively stained with Alizarin Red S staining, D-PDLSCs formed fewest mineralized nodules among three groups (Fig. 1A). Furthermore, Real-time PCR and western blot analyses showed that the expression of osteoblast specific gene run-related gene 2 (RUNX2) in D-PDLSCs was much lower than those in H-PDLSCs and P-PDLSCs following 14 days of osteogenic induction (p < 0.05) (Fig. 1C).

When PDLSCs were induced for 4 weeks for adipogenic differentiation, oil globules were formed with accumulation of lipid-rich vacuoles within cells, as confirmed by Oil Red O staining. The H-PDLSCs formed significantly more oil globules (p < 0.05), but no significant difference was found between P-PDLSCs and D-PDLSCs (Fig. 1B). Additionally, Real-time PCR and western blot analyses showed the expression levels of adipogenic key gene peroxisome proliferator-activated receptor gamma (PPARγ2) in P-PDLSCs and D-PDLSCs was lower as compared with H-PDLSCs following adipogenic induction, but there is no statistical difference for PPARγ2 gene expression between P-PDLSCs and D-PDLSCs (Fig. 1D).

### DKK1 could reverse decreased osteogenesis of D-PDLSCs

**Figure 2:**
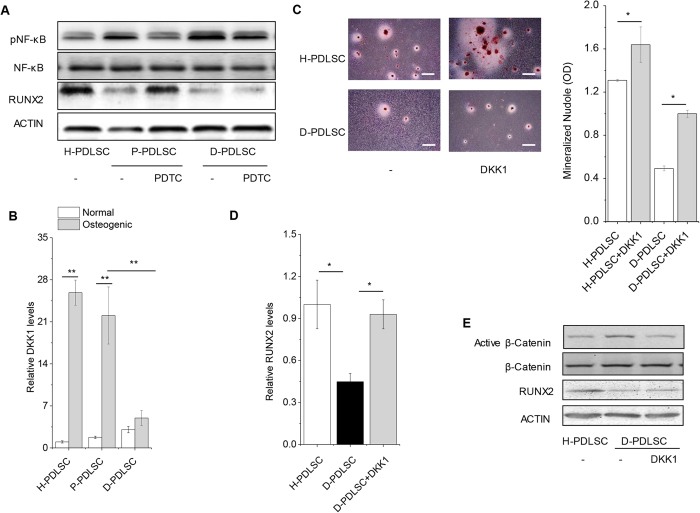
Inhibiting WNT signaling with DKK1 promoted osteogenic differentiation of D-PDLSCs. (**A**) To examine whether NF-κB signaling mediated impaired osteogenic potential of D-PDLSCs, we treated D-PDLSCs with NF-κB inhibitor PDTC. H-PDLSCs, P-PDLSCs and P-PDLSCs with PDTC were as control. The activation of NF-κB (phosphorylated p65, pNF-κB), RUNX2 and ACTIN was examined by western blot analysis. (**B**) H-PDLSCs, P-PDLSCs and D-PDLSCs were induced 7 days by osteogenic medium. The expression of DKK1 was examined by Real-time PCR (n = 4). (**C**) Quantification of Alizarin Red staining. PDLSCs for staining were induced to osteogenic differentiation for 28 days (n = 5), bar = 200 μm. (**D**,**E**) Real-time PCR and western blot analysis of the osteoblast marker gene (RUNX2, normalized to β-actin) on day 7 (n = 3). Active β-catenin which indicates WNT signaling is activated was examined by western blot analysis. Data (±SD) are representative of two (**D**) or three (**B**,**C**) independent experiments. Student’s t test was performed to determine statistical significance (*p < 0.05, **p < 0.01).

It was reported that inhibition of NF-κB could increase osteogenesis of PDLSCs from periodontitis^14^. The question is whether we could reverse the impaired osteogenic potential of D-PDLSCs through modulating NF-κB signaling. Indeed, activity of NF-κB was higher in P-PDLSCs and PDTC that inhibitor of NF-κB pathway could reverse impared expression of RUNX2 with blocking NF-κB signaling in P-PDLSCs (Fig. 2A). Though NF-κB signaling was also activated in D-PDLSCs, PDTC could not promote RUNX2 expression in D-PDLSCs (Fig. 2A).

We previously found that DKK1 could promote osteogenic differentiation of PDLSCs through regulating β-catenin^3^. To assess DKK1 of PDLSCs in response to inflammatory and diabetic stimulation, we determined the level of DKK1 by Real-time PCR analysis during a period of 7 days. H-PDLSCs and P-PDLSCs expressed the high level DKK1 with culturing in osteogenic medium, but its expression was not increased in D-PDLSCs (Fig. 2B). To directly investigate whether low expression of DKK1 results in impaired osteogenesis in D-PDLSCs, we cultured H-PDLSCs and D-PDLSCs in osteogenic differentiation medium with DKK-1 at 50 ng/ml. Interestingly, quantitative analysis suggested that the incubation of both H-PDLSCs and D-PDLSCs with DKK1 increased the formation of mineralized nodules (p < 0.05) (Fig. 2C). DKK1 could increase expression of RUNX2 through inhibiting active β-catenin in D-PDLSCs (Fig. 2D,E).

### AGEs could aggravate impaired osteogenesis of PDLSCs in inflammatory microenvironments *in vitro*

**Figure 3:**
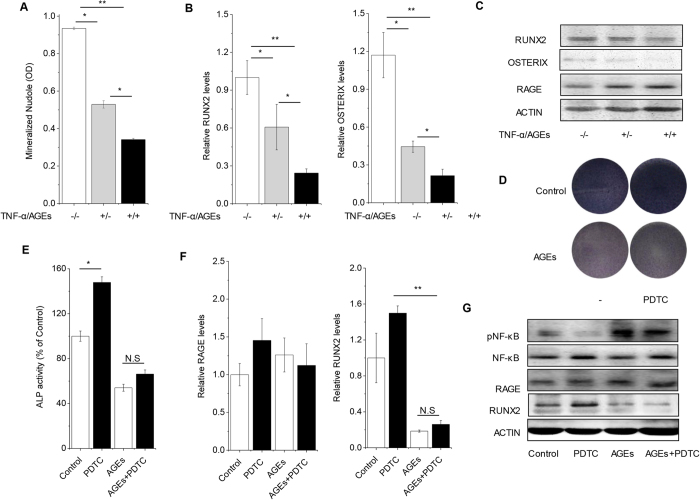
Effects of AGEs on osteogenesis of PDLSC *in vitro*. (**A**) PDLSCs were treated with TNF-α and AGEs, which mimics the PDLSCs from periodontitis with diabetes mellitus persons. Quantification of Alizarin Red staining was examined when PDLSCs with TNF-α and AGEs were induced in osteogenic differentiation for 28 days (n = 3). (**B**) The expression of osteoblastic gene RUNX2 and OSTERIX was examined by Real-time PCR on day 7 (n = 3). (**C**) The expression of RAGE and osteoblastic gene were examined by western blot analysis on day 7. (**D**,**E**) PDLSCs were treated with AGEs and NF-κB inhibitor PDTC. The ALP activity staining were quantified on day 7 (n = 4). (**F**) The expression of RAGE and osteoblastic gene were examined by Real-time PCR on day 7 (n = 3). (**G**) The activation of NF-κB (phosphorylated p65, pNF-κB), RUNX2 and RAGE was examined by western blot analysis. Data (±SD) are representative of two (**E**) or three (**A**,**B**,**F**) independent experiments. Student’s t test was performed to determine statistical significance (*p < 0.05, **p < 0.01).

Both P-PDLSCs and D-PDLSCs were isolated from periodontal tissues with inflammation, but osteogenic potential of D-PDLSCs could not be regulated by inflammatory related pathway-NF-κB. To find out what mediate the character of D-PDLSCs, we examined the effect of AGEs on PDLSCs with inflammatory microenvironment *in vitro*. As reported before, TNF-α decreased mineralized bone matrix formation of PDLSCs. Real-time RT-PCR and Western blot analysis indicated osteoblasts marker genes RUNX2 and OSTERIX were reduced by TNF-α. Furthermore, AGEs aggravated the effect of TNF-α on PDLSCs (Fig. 3A–C). Quantitative analysis suggested that the administration of AGEs in inflammatory microenvironment decreased the formation of mineralized nodules (p < 0.05). In parallel, Real-time PCR and western blot analyses showed the expression levels of osteoblast specific genes such as RUNX2 and OSTERIX in PDLSCs declined in response with AGEs that presented in the inflammatory cultures. Interestingly, the expression of RAGE which is binding AGEs was increased in inflammatory cultures but not affected by AGEs (Fig. 3C).

Furthermore, osteogenic potential of PDLSCs with AGEs treatment declined significantly in terms of ALP staining and ALP quantitation, and was not rescued by PDTC (Fig. 3D,E). AGEs could activate NF-κB signaling which was blocked by PDTC, and inhibit expression of RUNX2 that could not be rescued by PDTC. Along with effect of PDTC on osteogenic potential, expression of RAGE was also not affected by PDTC (Fig. 3F,G).

### DKK1 could inhibit effect of AGEs in PDLSCs and promote D-PDLSCs bone formation *in vivo*

**Figure 4:**
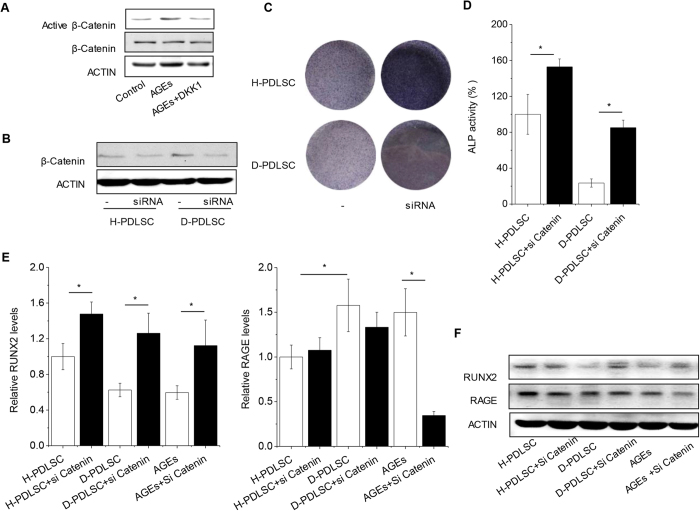
Inhibition of WNT could rescue the osteogenic differentiation of D-PDLSCs and PDLSCs with AGEs. (**A**) Western blot analysis of active β-Catenin in PDLSCs with AGEs were examined after DKK1 treatment. (**B**) The effect of β-Catenin siRNA was checked by western blot in H-PDLSCs and D-PDLSCs. (**C**,**D**) ALP activity was examined in H-PDLSCs and D-PDLSCs after treatment with siRNA for β-Catenin (n = 4). (**E**,**F**) Real-time RT-PCR and western blot analysis of the RUNX2 and RAGE on day 7 (n = 3). Data (±SD) are representative of three (**C**,**D**) independent experiments. Student’s t test was performed to determine statistical significance (*p < 0.05, **p < 0.01).

To confirm the role of WNT signaling regulated by DKK1 in D-PDLSCs, we used siRNA of β-catenin in our study to modulate WNT signaling. Active β-catenin was increased in D-PDLSCs or in H-PDLSCs with AGEs after culturing in osteo-inductive medium, and was reduced by DKK1 (Figs 2E and 4A). Indeed, β-catenin knocked down promoted osteogenic differentiation of H-PDLSCs and D-PDLSCs, which was indicated by ALP staining and ALP activity quantitation (Fig. 4B–D). Expression of RUNX2 was also increased by catenin siRNA in D-PDLSCs or PDLSCs with AGEs (Fig. 4E,F). We also found that the down-regulated expression of RAGE by β-catenin siRNA was only in H-PDLSCs treated with AGEs (Fig. 4E,F).

**Figure 5:**
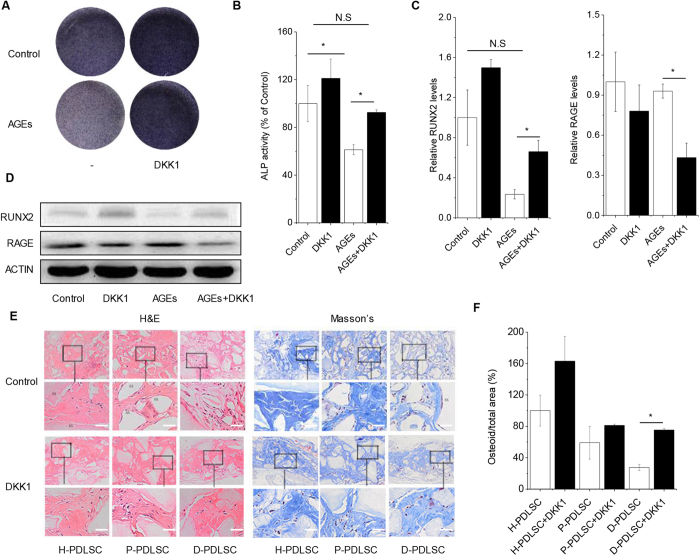
DKK1 rescued osteogenic potential of PDLSC impaired by AGEs *in vitro* and promoted bone formation of D-PDLSCs *in vivo*. (**A**,**B**) PDLSCs were treated with AGEs and WNT inhibitor DKK1 and induced in osteogenic medium for 7 days. The ALP activity staining were quantified on day 7 (n = 3). (**C**,**D**) Real-time RT-PCR and western blot analysis of the RUNX2 and RAGE on day 7 (n = 3). (**E**,**F**) H&E and Masson’s trichrome staining were performed 10 weeks after implantation, bar = 100 μm. Bone-like tissue was quantified as total osteoid volume per total volume by Masson’s staining. Four implants per treatment were engrafted into mice, and three sections of each implant were quantified to minimize variations within the implants (HA = hydroxyapatite, n = 4 per group). Data (±SD) are representative of two (**F**) or three (**B**,**C**) independent experiments. Student’s t test was performed to determine statistical significance (*p < 0.05, **p < 0.01).

In parallel, impaired osteogenic potential of PDLSCs with AGEs could also be reversed by DKK1. Interestingly, DKK1 could not only rescue osteogenic potential but also reduce expression of RAGE (Fig. 5A–D). To study the long-term effect of DKK1 in PDLSCs, we performed ectopic bone formation of PDLSCs *in vivo*. H-PDLSCs, P-PDLSCs and D-PDLSCs treated with DKK1 were loaded on a hydroxyapatite-tricalcium phosphate scaffold and implanted subcutaneously in NOD/SCID mice. After 10 weeks, bone-like tissue was increased 2.3-fold in the D-PDLSCs implants treated with DKK1 compared with D-PDLSCs (Fig. 5E,F). In contrast, DKK1 treated H-PDLSCs and P-PDLSCs only slightly enhanced bone formation by about 20% compared with H-PDLSCs and P-PDLSCs separately. These results suggest that DKK1 indeed promotes osteoblast differentiation of D-PDLSCs *in vitro and in vivo*.

## Discussion

PDLSCs are known for their stemness in local oral tissues by regenerating periodontal tissues, which leads to a favorable treatment for periodontal defect in periodontitis^16^,^17^. In this study we found that change of osteogenic potential of PDLSCs was associated with diabetic milieu. AGEs inhibited osteogenic potential of PDLSCs and increased impaired effect of TNF-α on PDLSCs *in vitro*. Inflammatory pathway NF-κB signaling primarily responded to inflammatory stimulation was uncoupled with diabetic milieu or AGEs treatment *in vitro*. Notably, DKK1 inhibiting canonical WNT pathway could rescue impaired osteogenic potential of D-PDLSCs.

PDLSCs have been identified as an important factor of periodontitis progression, as they have been found to regenerate the complex system of tooth-supporting apparatus (i.e., the periodontal ligament, alveolar bone and root cementum)^18^. Though we previously had demonstrated inflammatory effect on PDLSCs, we still want to understand whether simple periodontitis and complicate periodontitis had the same effect on PDLSCs. Four years ago, we had reported that different character of age matched donors for H-PDLSCs and P-PDLSCs^3^,^15^, and found DKK1 have the similar effect on H-PDLSCs and P-PDLSCs. DKK1 might rescue osteogenic potential of P-PDLSCs. Interestingly, compared with simply periodontitis, the altered inflammatory microenvironment induced by diabetes mellitus leads to reduced lower osteogenic potential but no significantly adipogenic changes. However, we could not completely exclude the aging effect on PDLSCs, since media age in healthy donors for generating H-PDLSCs was much younger (17-years) than patients isolated for D-PDLSCs, and 12 year younger than patients for P-PDLSCs. In clinic, it was very hard to get D-PDLSCs from young donors and H-PDLSCs from old donors. It seems that we could not exclude effect of age on osteoblastogenesis between H-PDLSCs and D-PDLSCs. However, the age of P-PDLSCs and D-PDLSCs of donors was similar. The age between the two groups are basically matched. Additionally, In this study, we treated H-PDLSCs with AGEs *in vitro* to mimic D-PDLSCs, and found similar results from H-PDLSCs with AGEs and D-PDLSCs. These characteristics suggested that diabetes mellitus might have a unique effect on PDLSCs expected for aggravating periodontitis.

There has also been a growing awareness that advanced glycation end products (AGEs) are intimately linked to other complication of diabetes mellitus^7^. Nuclear factor-kappaB (NF-κB) is well studied in inflammation and diabetes by many different groups^19^,^20^,^21^,^22^, and our recent report shows that NF-κB may help in improving stem cell-mediated inflammatory disease therapy in periodontal tissues^14^. However, we could not rescue the differentiation potential of PDLSCs either from periodontitis with diabetes patients or in AGEs stimulation *in vitro* which mimic diabetes mellitus microenvironments. These results suggested there might be another signaling mediating the decreased osteogenic potential of D-PDLSCs or PDLSCs with AGEs treatment.

It is known that WNT/β-catenin pathway is related to the pathogenesis of several diseases. Many reports have suggested that WNT signaling could be a major modulator in diabetes^23^,^24^,^25^,^26^. Additionally, a recent clinical study shows that circulating sclerostin (an inhibitor of WNT signaling) is increased in type 2 diabetes mellitus patients^27^. However, it is still unclear that roles of WNT signaling in periodontitis with diabetes. In this study, we found that DKK1 blocking WNT signaling indeed promoted osteogenic differentiation of D-PDLSCs *in vitro and in vivo*. Interestingly, we saw DKK1 could also promote the mineralization of H-PDLSCs, and we also examined *in vivo* bone formation of H-PDLSCs with or without DKK1. The *in vivo* bone formation results suggested that DKK1 might promote osteogenesis of H-PDLSCs. However, we found the promotion of DKK1 on H-PDLSCs was less stable than other PDLSCs groups. It might be microenvironment influence clonal formation units of PDLSCs. As we all known, PDLSCs are kinds of MSCs. Several studies indeed have shown the activation of WNT/beta-catenin pathway could promote osteogenic differentiation of MSCs^28^,^29^,^30^,^31^. It was also suggested that high concentration WNT3a treatment could inhibit osteogenesis of MSCs^32^,^33^,^34^. Interestingly, we have found WNT signaling have completely opposite effect on MSCs from bone marrow and periodontal tissue, and the regulation might influence different inflammatory effect on MSCs from bone marrow and periodontal tissue^29^,^35^. Both WNT3a and LiCl could activate WNT/beta-catenin pathway, and inhibit osteogenic differentiation of MSCs from periodontal tissue^36^. It was suggested PDLSCs expressed MSCs markers, and PDLSCs might be another MSCs in periodontal tissue. We supposed the difference might be related to different clonal formation units in MSCs. For this reason, the mechanism, by which WNT signaling activation suppresses osteoblastic differentiation in MSC from other tissue, need further investigate.

In conclusion, we found that the function of PDLSCs from periodontitis with diabetes mellitus patients was impaired, which was different with PDLSCs from only periodontitis patients. WNT signaling inhibitor treatment appeared to cause increased bone formation as well as partly regulate AGEs-RAGE axis, suggesting a possible mechanism by which AGEs induces PDLSCs dysfunction in our clinical cell model. The ultimate goal of treating periodontitis patients with diabetes mellitus is to rescue the defect of PDLSCs that are believed to regenerate periodontal tissues. Considering that inhibition of WNT signaling improves function of PDLSCs, as we reported in this study, DKK1 or a small molecule that mimic its function may help PDLSCs server as a potential agent to treat periodontitis patients with diabetes mellitus.

## Methods

### Subjects

Periodontitis patients without diabetes mellitus were aged from 30 to 70 years with a mean age of 56.95 ± 11.3 years, and The diagnosis of periodontitis met the following condition: (1) with over 20 teeth, probing depth(PD) over 5 mm, more than 30% teeth with attachment loss (AL) over 4 mm, or over 60% teeth with PD > 4 mm and AL > 3 mm; (2) without periodontal treatment in the last 6 months; (3) without antibiotics or non-steroidal anti-inflammatory drugs administered in the last 3months; (4) without serious systemic diseases and complications. Patients with systemic inflammatory diseases (rheumatoid arthritis, etc.), blood disease, liver damage, kidney disease or trauma were excluded.

Periodontitis patients with diabetes mellitus were aged from 47 to 76 with a mean age of 60.15 ± 8.9. The diagnosis of periodontitis met the condition shown as above, and the diagnosis of diabetes mellitus met the following criteria: (1) the diagnosis of diabetes mellitus over 1 year; (2) good glucose control with fasting blood glucose <7.0 mmol/L and HbA1c between 6.5%and 7.5%; (3) no medication changes in the last 3 months; (4) No smoke; (5) without severe complications.

The normal control group consisted of 10 healthy male adults, aged from 36 to 50 years with a mean age of 42.8 ± 5.1 years. Human third molars without caries, inflammation, or periodontitis were extracted for impaction reasons from 10 systemically healthy donors at the department of Endocrinology of affiliated hospital and dental hospital of Zunyi Medical College, Zunyi, China. For the aged donors whose third impacted molars were not removed in the correct stage, the extraction of teeth was based on complete oral health examinations showing that the corresponding teeth were harmful to the donors’ oral health and must be removed. A total of 20 patients with periodontitis and 20 periodontitis with type 2 diabetes mellitus patients were enrolled from the department of Endocrinology of affiliated hospital and dental hospital of Zunyi Medical College. Informed consents were obtained from all subjects. This study was approved by the Ethics Committee of Zunyi Medical College. All the methods were carried out in accordance with the approved guidelines.

Additional information of teeth condition and approach of teeth: Please see Supplemental Methods.

### PDLSCs isolation and culture

We isolated and cultured PDLSCs from the periodontal ligament tissue of 20 periodontitis patients and 20 periodontitis with DM and 10 healthy donors using the limiting dilution technique. All of cell lines are identified by flow cytometry to analyze the cell surface markers and by multi- differentiation experiment. All of cell lines expressed mesenchymal stem-cell markers and possess similar characteristics in H-PDLSCs, P-PDLSCs or D- PDLSCs. Passages of 2–4 were used in our experiments. Therefore, cell number was limited for one cell line, and one cell line could not be performed all of the experiments, especially cell lines in D-PDLSCs group. As a result, we used 3–5 different lines for each experiment by random-selection method. The results should be able to represent general characteristic of H-PDLSCs, P-PDLSCs or D- PDLSCs.

The experimental guidelines dealing with human PDLSCs were approved by the institutional review board at our institute (School of Stomatology, The Fourth Military Medical University, Ethics Committee). Primary human PDLSCs were cultured as described previously^15^, and and protocols were in accordance with the approved guidelines. In brief, we gently washed the teeth with sterile phosphate-buffered solution (PBS) (Boster, Wuhan, China) and separated PDL tissues from the middle part of the root surface. PDLSCs were isolated using the limiting dilution technique and cultured in α-minimum essential medium (α-MEM; Gibco, Grand Island, NY, USA) supplemented with 10% fetal bovine serum (FBS; Sijiqing, Hangzhou, China), 0.292 mg/mL glutamine ((Invitrogen, Grand Island, NY, USA), 100 U/mL penicillin, 100 mg/mL streptomycin (Gibco) at 37 °C in a humidified atmosphere of 5% CO2. After 2–3 weeks in culture, the single cell-derived clones were harvested and mixed together. Multiple colony-derived PDLSCs from normal control group (H-PDLSCs), periodontitis patients without diabetes mellitus (P-PDLSCs) and periodontitis patients with diabetes mellitus (D-PDLSCs) at 2–4 passages were used in our experiments. Each experiment repeated at least three times. Tumor necrosis factorα (TNF-α) and DKK1 were purchased from Pepro-Tech (Rocky Hill, NJ, USA). To mimic diabetes microenvironments *in vitro*, AGE-BSA (BioVision, Milpitas, CA, USA) was used to treat H-PDLSCs at different concentrations (0, 1, 10, 100, and 200 ng/mL). We transfected PDLSCs with siRNA targeting β-catenin or control siRNA that has been tested to not lead to the specific degradation of any known cellular mRNA (GenePharma, Shanghai, China) using Lipofectamine RNAiMAX in Opti-MEM (Invitrogen) according to the protocol recommended by the manufacturer.

### Osteogenic and adipogenic differentiation

The third-passage of H-PDLSCs, P-PDLSCs and D-PDLSCs were seeded into at a density of 1 × 105 each well of a six-well plate. At 80% confluent, PDLSCs were cultured in osteogenic medium (α-MEM supplemented with 10% FBS, 5 mM L-glycerophosphate (Sigma, St Louis, MO, USA), 100 nM dexamethasone (Sigma), and 50 ug/ml ascorbic acid. After 4-week induction, the samples were fixed with 4% polyoxymethylene for 30 min; 0.1% Alizarin red staining (Sigma) was performed to determine mineralization as previously reported.

At 100% confluent, PDLSCs were cultured in adipogenic medium (α-MEM supplemented with 10% FBS, 2 μM insulin (Sigma), 0.5 mM isobutyl-methylxanthine (IBMX; Sigma), 0.5 μM hydrocortisone and 1 mM dexamethasone (Sigma)). The control group was cultured in a-MEM plus 10% FBS. The medium was changed every two days for 3 weeks. The experiment was repeated three times. After 3 weeks of induction, the cells growing under adipogenic conditions were washed twice with PBS and fixed in 70% ethanol for 15 min. Oil red O staining (Sigma) was performed as previously reported.

### Alkaline phosphatase (ALP) activity assay

For quantitative analysis of alkaline phosphatase (ALP) activity, single-cell suspensions of H-PDLSCs, P-PDLSCs and D-PDLSCs were seeded at a density of 3 × 103 cells/well into 96-well plates and cultured in α-MEM at 37 °C in a humidified atmosphere of 5% CO2, and 95% air. At 24 hours the media was changed to the osteogenic induction media, after 3-, 5-, and 7-day *in vitro* culture, the ALP activity of each cell was detected with a commercially available assay kit (Zhongsheng, Beijing, China). In brief, cells were washed 3 times in PBS and incubated in Triton X-100 (3 ml/l in PBS) for 4 hours. 100 μl of pnitrophenol phosphate substrate solutions was added to each well and the cells were incubated for 40 min at 37 °C, and then the reaction was stopped with 0.1 M NaOH. The OD value was measured by spectrophotometer at 405 nm using a microplate reader. The experiment was repeated three times.

### Quantitative real time-polymerase chain reaction (Real time-PCR)

Total RNA was isolated from PDLSCs of three groups using the Trizol reagent (Invitrogen). Approximately 1 μg of total RNA was converted to cDNA by using the Super Script First Strand Synthesis kit (Invitrogen). The Real time-PCR reactions were performed using the QuantiTect SYBR Green PCR kit (Toyobo, Osaka, Japan) and the CFX96 Touch Real-Time PCR Detection System (Bio-rad, Hercules, CA, USA). Two independent experiments were performed for each reaction in triplicate. The primer sequences used in the experiment were listed in Supplementary table 2.

### Protein isolation and western blot analysis

Total proteins were extracted from the cells by lysis in RIPA buffer (10 mM Tris-HCl, 1 mM EDTA, 1% sodium dodecyl sulfate, 1% Nonidet P-40, 1:100 proteinase inhibitor cocktail, 50 mM β-glycerophosphate, 50 mM sodium fluoride). We determined the protein concentration in the extracted lysates by measuring the absorbance at 595 nm by using a protein assay solution (Bio-Rad, Hercules, CA, USA). Aliquots of 20–50 μg of the cell lysates per sample were separated by 10% sodium dodecyl sulfate-polyacrylamide gel electrophoresis (SDS-PAGE) and then transferred to a polyvinylidene fluoride (PVDF) membrane (Bio-Rad). The membranes were blocked with 5% milk for 2 hours and then incubated with primary antibodies overnight at 4 °C The immunocomplexes were incubated with horseradish peroxidase-conjugated anti-rabbit or anti-mouse IgG antibodies (Boster). Immunodetection was performed using the Western-Light chemiluminescent detection system (Peiqing, Shanghai, China). The experiment was repeated three times.

### *In vivo* bone formation assay

All of the procedures that involved animals were approved by the Animal Use and Care Committee of the Fourth Military Medical University (license number: SCXK 2007-007). For a single transplant complex, the PDLSCs were treated with DKK1 (100 ng/ml) as described above, and then cultured for 3 days. Approximately 5.0 × 10^6^ cells were mixed with 40 mg of CBB powders (Research and Development Center for Tissue Engineering, Xi’an, China) and implanted into subcutaneous pockets on the backs of the 8-week NOD/SCID mice (Fourth Military Medical University). As a control, PDLSCs from the same sources that had been treated with DMSO were implanted into the other side of the same host. The implants were removed after 8 weeks, fixed in 4% paraformaldehyde, decalcified in EDTA (pH 7.0), and embedded in paraffin. The samples were cut into 5-μm sections and stained with H&E or Masson’s Trichrome staining.

### Statistical analysis

All values were expressed as mean ± standard deviation. Statistical significance was assessed by an independent samples t test with SPSS 16.0 software (SPSS, San Rafael, CA, USA). Statistical significance was determined at p < 0.05. All data acquisition and analyses were performed blindly.

## Additional Information

**How to cite this article**: Liu, Q. *et al*. DKK1 rescues osteogenic differentiation of mesenchymal stem cells isolated from periodontal ligaments of patients with diabetes mellitus induced periodontitis. *Sci. Rep*. **5**, 13142; doi: 10.1038/srep13142 (2015).

## Supplementary Material

Supplementary Information

Supplementary Table S2
