# Supplementary material for: DKK1 rescues osteogenic differentiation of mesenchymal stem cells isolated from periodontal ligaments of patients with diabetes mellitus induced periodontitis
Source: Sci Rep. 2015 Aug 17;5:13142. doi: 10.1038/srep13142 (PMC4538385; doi:10.1038/srep13142)
Supplement: Supplementary Table S2 [file srep13142-s2.doc]

**DKK1 rescues osteogenic differentiation of mesenchymal stem cells isolated from periodontal ligaments of patients with diabetes mellitus induced periodontitis**

Qi Liu^1, 2, 4, a^, Cheng-Hu Hu^2, 5, a^, Cui-Hong Zhou^2, 5, a^, Xiao-Xia Cui^4^, Kun Yang^4^, Chao Deng^4^, Jia-Jia Xia^4^, Yan Wu^4^, Lu-Chuan Liu^1*^, Yan Jin^2, 3, 6*^

^1^ Department of stomatology, Daping hospital, Research institute of Field surgery, Third military medical university, Chongqing, 400042, China

^2^ State Key Laboratory of Military Stomatology, Center for Tissue Engineering, School of Stomatology, Fourth Military Medical University, Xi’an, Shaanxi 710032, China

^3^ Research and Development Center for Tissue Engineering, Fourth Military Medical University, Xi’an, Shaanxi 710032, China

^4^ Department of Periodontology, Stomatological hospital, Zunyi medical college, Zunyi, Guizhou 563003, China

^5^ Xi’an Institute of Tissue Engineering & Regenerative Medicine, Shaanxi 710032, China

^6^ Department of Oral Histology and Pathology, School of Stomatology, Fourth Military Medical University, Xi’an, Shaanxi 710032, China

^a^ These authors contribute equally to this work

^*^ Corresponding authors:

**Supplementary Methods**

**Approach of dealing with teeth**

If one donor had more than two teeth that could be extracted, two teeth were randomly included in the present investigation. If a tooth was destroyed or contaminated during or after the surgery, it was automatically excluded from the study. However, if the cells failed to grow from tissue samples for nontechnical reasons, the donor information was included into final statistical analysis. Each tooth was studied independently, even if two were from the same donor.

**The Standard of Caries**

Normal: no caries;

Enamel caries: caries deep into enamel, but not to penetrate into dentin;

Superficial caries of dentin: caries penetrate into superficial dentin;

Deep caries: caries penetrate into deep dentin, but not to arrive at dental pulp;

Residual crown: large area caries of crown;

Residual root: caries lead to deficiency of crown, and caries arrive at root;

Pulpitis: caries reach at dental pulp and lead to inflammatory pulp;

Devital tooth: caries reach at dental pulp and lead to inactivity of pulp;

**The Standard of Abrasion Degree**

Negative: no abrasion;

Mild: the characteristics of these enamels lose and the shape of dental cervix change in a minute scale;

Moderate: enamel lose off dentin exposure but does not exceed l/3 enamel and dentin which on incisal lose, but not yet exposed secondary dentin and pulp; the depth of tooth cervix defect is 1-2mm;

Severe: enamel is gone, pulp or the secondary dentin exposure, dentin exposure exceed 2mm.

**The Standard of Tooth Mobility Degree**

The range of tooth mobility:

I: degree of severity based on the loose range does not exceed 1mm;

II: degree is 1-2mm;

III: degree exceed 2mm.

The direction of tooth mobility:

I: degree of severity based on the mobility only of lip or buccal;

II: degree expand to mesial and distal shape;

III: degree is II degree with vertical direction.

**The Standard of Calculus Index**

Negative degree(-): no soft dirt and dental calculus;

I degree(+): A little soft dirt or calculus, but does not exceed l/3;

II degree(++): the dental calculus did not exceed the crown surface 1/3 and there are a small number of sub-gingival calculus;

III degree(+++): the dental calculus does not exceed the crown surface of 2/3 and there are more sub-gingival calculus.

**Supplementary Table 1**

**General characteristics of teeth condition and information of participants in this study.**

A total of 13 healthy teeth from 10 donors, 32 teeth from 20 periodontitis donors and 35 teeth from periodontitis with diabetes were involved in the present study. Periodontal ligament tissues from each tooth were independently collected for cell culture and divided into H-PDLSCs, P-PDLSCs and D-PDLSCs groups according to the donors.
